# Laparoscopic versus open loop ileostomy reversal: A systematic review and meta-analysis

**DOI:** 10.1016/j.sipas.2023.100161

**Published:** 2023-03-23

**Authors:** Tyler McKechnie, Léa Tessier, Tharani Anpalagan, Megan Chu, Yung Lee, Kathleen Logie, Aristithes Doumouras, Nalin Amin, Dennis Hong, Cagla Eskicioglu

**Affiliations:** a Division of General Surgery, Department of Surgery, McMaster University, Hamilton, ON, Canada; b Michael G. DeGroote School of Medicine, McMaster University, Hamilton, ON, Canada; c Division of General Surgery, Department of Surgery, St. Joseph Healthcare, Hamilton, ON, Canada

**Keywords:** Bowel resection, Colorectal surgery, Loop Ileostomy, Loop ileostomy reversals, Laparoscopy

## Abstract

**Background:**

Loop ileostomies (LIs) are used for temporary fecal diversion to protect downstream colorectal anastomoses. Standard operative approach for LI reversal has been through an open technique. Recently, laparoscopic LI reversal has been employed and studied. The aim of this systematic review was to compare laparoscopic and open LI reversal.

**Methods:**

Medline, Embase, and CENTRAL were systematically searched. Articles were included if they compared rate of postoperative morbidity and/or length of stay (LOS) in patients undergoing laparoscopic or open LI reversal. Pairwise meta-analyses using inverse variance random effects was performed. The Grading of Recommendations, Assessment, Development, and Evidence (GRADE) approach was conducted to assess quality of evidence.

**Results:**

From 410 citations, four observational studies with 213 patients undergoing laparoscopic LI reversal and 176 patients undergoing open LI reversal met inclusion. Patients in the laparoscopic group had significantly shorter LOS (MD -0.39, 95%CI -0.73 to -0.04, *p* = 0.03). Laparoscopic and open LI reversal were comparable in postoperative morbidity, aside from a decrease of superficial surgical site infection (sSSI) with the use of laparoscopy (OR 0.22, 95%CI 0.07 to 0.71, *p* = 0.01). Operative time was not significantly different between groups (MD 11.91, 95%CI -1.87 to 25.70, *p* = 0.09). The GRADE quality of evidence was low to very low.

**Conclusions:**

This review presents low quality evidence that laparoscopic LI reversal is a feasible approach that may reduce postoperative LOS and sSSI compared to open LI reversal without increasing operative time. Future prospective comparative studies are required to confirm the findings of the present review.

## Introduction

Sphincter-sparing surgical approaches to rectal pathology have become increasingly sophisticated and implemented into clinical practice.^1^^,^^2^ Ultra-low anterior resections (LAR), trans-anal total mesorectal excision (TaTME), and robotic surgery have facilitated colorectal resection with adequate margins deep in the pelvis and subsequent reconstruction with low colorectal or colo-anal anastomoses.[2], [3], [4] Following low rectal anastomoses, loop ileostomies (LIs) are often relied upon for temporary fecal diversion in order to protect the downstream anastomoses and prevent against the potential consequences of anastomotic leak.^5^^,^^6^

The interval between LI creation and reversal varies significantly and is dependent on indication. Some centres have reported reversal within two weeks of creation with good outcomes, whereas patients undergoing adjuvant chemotherapy for rectal cancer can live with LIs for more than a year.[7], [8], [9] Traditionally, LI reversal has been done through an open approach whereby a circumferential dissection beginning at the level of the skin is used to free the small bowel loop forming the LI, followed by creation of an extracorporeal anastomosis.^10^ Recently laparoscopic LI reversal has been described and studied.^11^^,^^12^ This approach has been advocated for as surgeon experience and comfort with laparoscopic surgery has increased and patients have become increasingly obese.^11^^,^^13^ In brief, this involves intracorporeal transection of the small bowel followed by intracorporeal reconstitution of gastrointestinal continuity. The remnants of the in-situ LI are then excised via an open circumferential dissection from the level of the skin. Subsequent fascial and wound closure can proceed in a similar fashion to open LI reversal.

There have been few studies comparing open and laparoscopic LI reversal and equipoise remains regarding the benefits of one compared to the other.[12], [13], [14], [15] These data have yet to be synthesized. As such, the aim of this study is to systematically review and meta-analyze intraoperative and postoperative outcomes from studies comparing laparoscopic and open LI reversal.

## Materials and methods

### Search strategy

The following databases covering the period from database inception through July 2022 were searched: Medline, Embase, and Cochrane Central Register of Controlled Trials (CENTRAL). The search was designed and conducted by a medical research librarian with input from study investigators. Search terms included “loop ileostomy”, “reversal”, “laparoscopic”, “open”, and more (complete search strategy available in Appendix 1). References of published studies and gray literature were manually searched to ensure that all relevant articles were included. This systematic review and meta-analysis is reported in accordance with Preferred Reporting items for Systematic Reviews and Meta-Analyses (PRISMA).^16^ The study protocol was registered on the International Prospective Register for Systematic Reviews (PROSPERO) *a priori* (CRD42022357920). Local ethics review board approval was not required for this study.

### Study selection

Articles were eligible for inclusion if they compared laparoscopic and open LI reversal in length of stay and/or postoperative morbidity. Studies evaluating robotic LI reversal were eligible for inclusion. Relevant single-arm studies were excluded. Studies including less than 10 patients and patients undergoing urgent/emergent reversal were excluded. Studies were not discriminated on the basis of language, year of publication, or index surgery necessitating formation of a diverting LI. Conference proceedings, opinions, case reports, reviews, meta-analyses, letters to editors and editorials were excluded.

### Outcomes assessed

The primary outcome was postoperative LOS in days. LOS was defined as the time from the end of the index procedure (i.e., last suture/staple placed to close surgical incisions) to the time the patient left the hospital following their index procedure in all included studies.

Secondary outcomes included (1) 30-day postoperative morbidity; (2) return of bowel function postoperatively; (3) intraoperative outcomes; and (4) total inpatient healthcare cost. Return of bowel function outcomes included rate of postoperative ileus (POI), time to first flatus, time to first bowel movement, and time to resumption of oral intake. Postoperative morbidity was defined as any deviation from the expected postoperative course documented in patient medical records or database records. Postoperative morbidity included the following: anastomotic leak, intraabdominal abscess, superficial surgical site infection (sSSI), sepsis, hemorrhage, acute urinary retention (AUR), urinary tract infection (UTI), atelectasis, pneumonia, deep vein thrombosis (DVT), pulmonary embolism (PE), myocardial injury, and readmission to hospital. Intraoperative outcomes included operative time (in minutes) and estimated blood loss (EBL) (in milliliters (mL)).

### Data extraction

Two reviewers independently screened the systematically searched titles and abstracts using a standardized, pilot-tested form. Discrepancies that occurred at the title and abstract screening phases were resolved by inclusion of the study. At the full-text screening stage, discrepancies were resolved by consensus between the two reviewers. If the disagreement persisted, a third reviewer was consulted. Two reviewers extracted data into a data collection form designed *a priori.* The extracted data included study characteristics (e.g., author, year of publication, study design), patient demographics (e.g., age, gender, diagnosis, operation type, comorbidities), treatment characteristics (e.g., index procedure, neoadjuvant therapy, adjuvant therapy, duration of LI, operative time, and EBL), postoperative outcomes (e.g., LOS, postoperative mortality, postoperative morbidity, time to return of bowel function), and healthcare cost (i.e., total inpatient healthcare cost).

### Risk of bias assessment and certainty of evidence

Risk of bias for each of the included observational study was assessed using the Risk of Bias in Non-Randomized studies- of Interventions (ROBINS-I) assessment tool.^17^ Quality of evidence for estimates derived from meta-analyses were assessed by Grading of Recommendations, Assessment, Development, and Evaluation (GRADE).^18^ Two reviewers assessed study quality for each study. Discrepancies were discussed amongst the reviewers until consensus was reached.

### Statistical analysis

All statistical analysis and meta-analysis were performed on STATA version 14 (StataCorp, College, TX) and Cochrane Review Manager 5.3 (London, United Kingdom). The calculations and organization of results into a summary of findings table was done using the GRADEPro software.^19^ The threshold for statistical significance was set *a priori* at a *p* of <0.05. A pairwise meta-analysis was performed using a DerSimonian and Laird random effects model for all meta-analyzed outcomes. Pooled effect estimates were obtained by calculating the mean difference (MD) in outcomes for continuous variables and odds ratios (OR) for dichotomous variables along with their respective 95% confidence intervals (CI) to confirm the effect size estimation. In addition, mean and standard deviation (SD) was estimated for studies that only reported median and interquartile range using the method described by Wan et al.^20^ For studies that did not report standard deviation or interquartile range, we contacted the authors for missing data. Data was presumed to be unreported if no response was received from study authors within two weeks from the original contact point. Missing SD data were then calculated according to the prognostic method.^21^ A funnel plot for assessing publication bias was not used as this review contained less than 10 studies.^22^ Assessment of heterogeneity was completed using the inconsistency (I^2^) statistic. An I^2^ greater than 50% was considered to represent considerable heterogeneity.^23^ For outcomes that were reported in less than three studies, a systematic narrative summary was provided.^24^

## Results

### Study characteristics

**Fig. 1:**
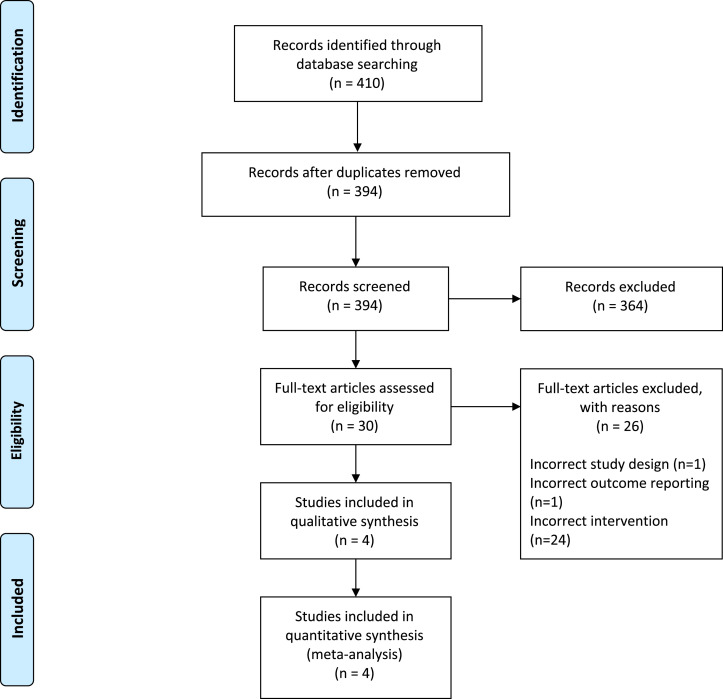
PRISMA Diagram – Transparent reporting of systematic reviews and meta-analysis flow diagram outlining the search strategy results from initial search to included studies.(1).

From 410 citations, four studies (all retrospective cohorts) with 213 patients undergoing laparoscopic LI reversal (40.8% female, mean age: 50.1) and 176 patients undergoing open LI reversal (43.8% female, mean age: 52.1) met inclusion criteria.[12], [13], [14], [15] A PRISMA flow diagram of the study selection process is illustrated in Fig. 1. Preoperative comorbidities were reported by three studies and included hypertension (*n* = 94, 28.6%), diabetes mellitus (*n* = 46, 14.0%), hypothyroidism (*n* = 12, 3.6%), coronary artery disease (*n* = 11, 3.3%), and more.[12], [13], [14] The mean duration patients had LIs for was 7.5 months in the laparoscopic group and 6.4 months in the open group. Index procedures were most commonly performed for colorectal malignancy (*n* = 145, 56.4%), inflammatory bowel disease (*n* = 89, 34.6%), and diverticular disease (*n* = 5, 1.9%). Included studies were conducted between 2015 and 2021. Detailed study characteristics are reported in Table 1.

**Table 1:** Study characteristics of included studies (N, number of patients; BMI, body mass index; SD, standard deviation; IBD, inflammatory bowel disease; UC, ulcerative colitis; CD, Crohn's disease; HTN, hypertension; CAD, coronary artery disease; CKD, chronic kidney disease; DM, diabetes mellitus; CHF, congestive heart failure; COPD, chronic obstructive pulmonary disease).

Study	N total	Arm	N	Mean age (years)	% Female	Mean BMI (SD)	Diagnosis/Indication for Initial Surgery	Comorbidities (%)
Wan, 2021	60	Laparoscopic	48	36.5 (18–70)*	25.0	20.2 (4.9)	CD- 48 (100)	–
		Open	12	39.8 (20–73)	33.3	20.3 (2.9)	CD- 12 (100)	–
Su, 2020	64	Laparoscopic	30	57.0 (51.3–63.5)⁎⁎	36.7	28.9 (28.5–29.8)	Colon Cancer- 1 (3.3)	HTN- 5 (16.7)
							Rectal Cancer- 29 (96.7)	CAD- 1 (3.3)
								CKD- 2 (6.7)
								DM- 3 (10)
		Open	34	60.5 (54.0–68.3)⁎⁎	29.4	28.8 (28.3–30.5)	Colon Cancer- 2 (5.9)	HTN- 9 (26.5)
							Rectal Cancer- 32 (94.1)	CAD- 1 (2.9)
								CKD- 3 (8.8)
								DM- 6 (17.6)
Sujatha-Bhasker, 2018	132	Laparoscopic	82	54.1 (14.4)	46.3	25.9 (4.9)	Colon cancer- 5 (6.1)	HTN- 26 (31.7)
							Rectal cancer- 53 (64.6)	CHF- 2 (2.4)
							UC- 11 (13.4)	COPD- 1 (1.2)
							Diverticular disease- 3 (3.7)	Smoker- 5 (6.1)
							Other- 10 (12.2)	DM- 13 (15.9)
								Steroid Use- 1 (1.2)
								Chemotherapy with 90 days- 27 (32.9) Bleeding disorder- 1 (1.2)
		Open	50	51.0 (14.3)	50.0	24.4 (4.4)	Colon cancer- 4 (8.0)	HTN- 15 (30.0)
							Rectal cancer- 19 (38.0)	COPD- 2 (4.0)
							UC- 15 (30.0)	Smoker- 4 (8.0)
							CD- 3 (6.0)	DM- 4 (8.0)
							Diverticular Disease- 2 (4.0)	Steroid Use- 2 (4.0)
							Other- 7 (14.0)	Chemotherapy with 90 days- 12 (24.0)
Young, 2015	133	Laparoscopic	53	52.2 (15.3)	49.1	27 (7.0)	–	CAD- 2 (3.8)
								HTN- 18 (34.0)
								DM- 9 (17.0)
								Hypothyroidism- 4 (7.6)
		Open	80	51.2 (18.1)	47.5	26.3 (6.3)	–	CAD- 7 (8.8)
								HTN- 21 (38.8)
								CKD- 1 (1.3)
								DM- 11 (13.8)
								Hypothyroidism- 8 (10.0)

⁎ =mean (range);.

⁎⁎ median (range).

### Treatment characteristics

**Fig. 2:**
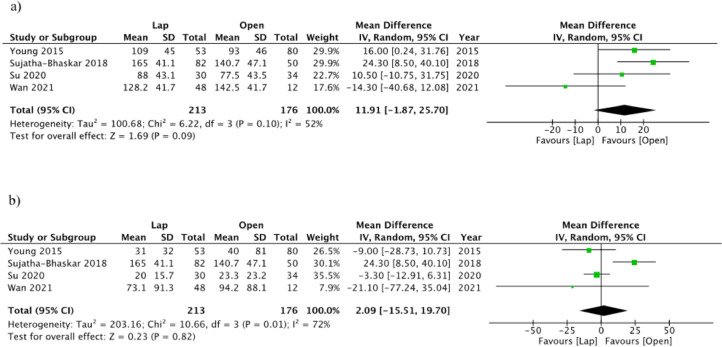
Operative Time (a) and Estimated Blood Loss (b) – Random effect meta-analysis comparing open and laparoscopic loop ileostomy reversal.

Treatment characteristics pertaining to both the index procedure as well as LI reversal are reported in Supplemental Table 1. Operative time was not significantly different between laparoscopic and open LI reversals (MD 11.91, 95%CI −1.87 to 25.70, *p* = 0.09, I^2^=52%) (Fig. 2a). In Sujatha-Bhaskar et al., the mean operative time required for laparoscopic LI reversal with intracorporeal anastomosis was significantly longer (172 min) compared to both open LI reversal (140.7 min) and laparoscopic LI reversal with extracorporeal anastomosis (157.6 min). In Young et al., the mean operative time in laparoscopic LI was 109 min versus 93 min in the open group. The other two studies showed no statistical difference in operative time between the two groups. Su et al. reported a median operative time of 88 min for laparoscopic LI and 77.5 minutes for open LI. Wan et al. reported a mean operative duration of 128.2 min and 142.5 min for laparoscopic LI and open LI, respectively. There was no significant difference in EBL between the two approaches (MD 2.09, 95%CI −15.51 to 19.70, *p* = 0.82, I^2^=72%) (Fig. 2b). Four of the included studies reported whether lysis of adhesions (LOA) was necessary at the time of reversal. In the laparoscopic group, 147 patients (69.0%) required LOA, compared to 72 patients (40.9%) in the open group (OR 2.46, 95%CI 0.77–7.89, *p* = 0.13, I^2^=82%). One study reported the type of closure at the stoma site. In the open LI group, 77.5% had pursed string closure, 6.3% had closure with staples and loose packing, and 7.5% were left open. In the laparoscopic group, these values were 88.7%, 0%, and 11.3%, respectively.

### Length of stay

**Fig. 3:**
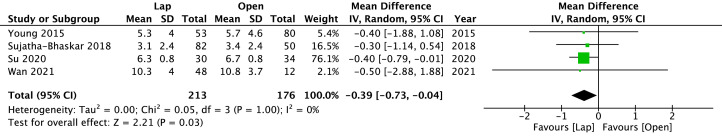
Length of Stay - Random effect meta-analysis comparing open and laparoscopic loop ileostomy reversal.

All included studies reported LOS data. On pooled analysis, LOS was significantly shorter in the laparoscopic group compared to the open group (MD −0.39, 95%CI −0.73 to −0.04, *p* = 0.03, I^2^=0%) (Fig. 3). Three of the four studies found no significant difference in length of stay between laparoscopic and open LI groups.^12^^,^^13^^,^^15^ In Sujatha-Bhaskar et al., patients who underwent laparoscopic LI reversal with intracorporeal anastomosis had a significantly shorter length of stay (52.1 h) compared to open (69.0 h) and LI reversal with extracorporeal anastomosis (69.6 h).^14^

### Postoperative outcomes

**Fig. 4:**
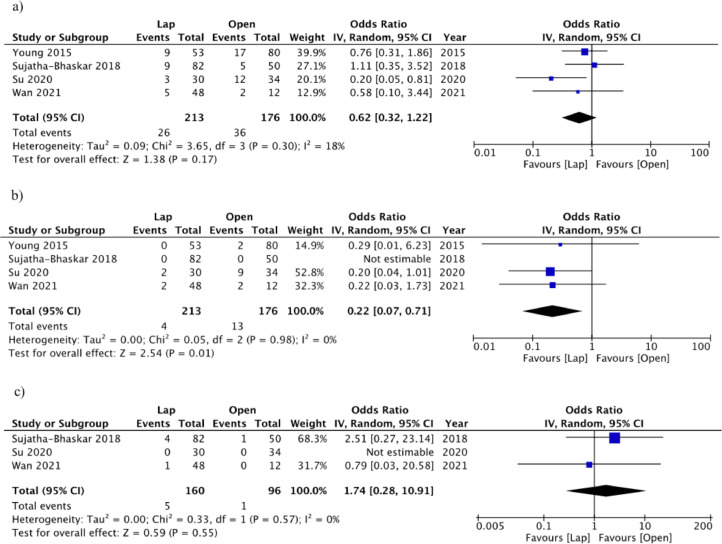
Postoperative Morbidity (a), Superficial Surgical Site Infection (b), and Postoperative Ileus (c) - Random effect meta-analysis comparing open and laparoscopic loop ileostomy reversal.

All included studies reported overall 30-day postoperative morbidity. There was no significant difference in postoperative morbidity between laparoscopic and open LI (OR 0.62, 95%CI 0.32 to 1.22, *p* = 0.17, I^2^=18%) (Fig. 4a). The laparoscopic LI group had significantly reduced sSSI compared to the open LI group (four studies; OR 0.22, 95%CI 0.07 to 0.71, *p* = 0.01, I^2^=0%) (Fig. 4b). Rates of postoperative morbidity per individual study are reported in Supplemental Table 2.

### Return of bowel function

Three of the included studies reported rates of POI. There was no significant difference in incidence of POI between groups (RR 1.74, 95% CI 0.28 to 10.91, *p* = 0.55, I^2^=0%) (Fig. 4c). Detailed return of bowel function data was reported in two of the included studies.^13^^,^^15^ Time to first flatus was significantly reduced in the laparoscopic LI reversal group in the observational study by Su et al. (2 days (IQR: 2–3 days) vs. 3 days (IQR: 2–4 days), *p* = 0.02).^13^ In the observational study evaluating patients with Crohn's disease undergoing LI reversal by Wan et al.*,* there was no significant difference between groups in terms of days to flatus (laparoscopic: 2.3d SD 0.8, open: 2.0d SD 0.7, *p*>0.05) or days to tolerance of soft diet (laparoscopic: 5.1d SD 3.9, open: 3.8d SD 1.4, *p*>0.05.^15^

### Cost

Two of the included studies reported healthcare expenditure data. Wan et al. found no significant difference between laparoscopic and open LI for the cost of the index admission following LI reversal (12,224.6 USD vs. 10,156.8 USD, *p*>0.05; converted from RMB to USD based on average 2021 exchange rate).^15^ Sujatha-Bhaskar et al. reported total healthcare costs, including costs associated with readmissions within 30-days, in US dollars, and found no significant difference between groups (laparoscopic: $10,761 (IQR $9934.50 - $12,901.50) vs. open: $10,386 (IQR $9127.00 - $13,855.00).^14^

### Risk of bias

Supplemental Figure 1 presents the risk of bias analyses according to the ROBINS-I for the included observational studies. The pooled risk of bias analysis according to the ROBINS-I is presented in Supplementary Figure 2. All of the included studies were at some risk of bias with regards to possible residual confounding, but otherwise were at low risk of bias across all other domains.

### Certainty of evidence

The GRADE certainty of evidence summary table is presented in Supplemental Figure 3. Overall certainty of evidence for LOS and sSSI outcomes were low. For the remaining meta-analyzed outcomes (i.e., 30-day postoperative morbidity, POI, operative time, EBL), overall certainty of evidence was very low. All outcomes were downgraded due to indirectness and imprecision. Variability in geographic location of study, indications for LI formation and reversal, and definitions of outcomes lead to serious concerns for inconsistency. Low event rates, small pooled sample sizes, and wide 95% confidence intervals lead to serious concerns for imprecision. There were no major concerns with risk of bias or inconsistency across the majority of studies.

## Discussion

LIs are frequently used for temporary fecal diversion to protect downstream colorectal or colo-anal anastomoses.^5^^,^^6^ Timing of reversal varies, and standard technique is via an open approach with an extracorporeal anastomosis. Recently, laparoscopic approaches to LI reversal have been developed and studied given the increasing expertise with minimally invasive surgery and the rising prevalence of obesity.^11^^,^^13^ This systematic review and meta-analysis pooled data from all previously published studies comparing laparoscopic and open LI reversal. Low certainty evidence demonstrated that postoperative LOS was reduced by almost half a day in patients undergoing laparoscopic reversal (MD −0.39, 95%CI −0.73 to −0.04, *p* = 0.03). Overall 30-day postoperative morbidity was not significantly different between groups, but incidence of postoperative sSSI was significantly reduced with the laparoscopic reversal according to low certainty evidence (OR 0.22, 95%CI 0.07 to 0.71, *p* = 0.01). Incidence of POI was not significantly different between the two groups according to very low certainty evidence (RR 1.74, 95% CI 0.28 to 10.91, *p* = 0.55). Intraoperative outcomes were also similar between the two approaches, with no significant differences in operative time (MD 11.91, 95%CI −1.87 to 25.70, *p* = 0.09) or EBL (MD 2.09, 95%CI −15.51 to 19.70, *p* = 0.82) according to very low certainty evidence.

The contemporary surgeon has a sophisticated skill set in laparoscopy and has increasing comfort applying these skills to a wide range of operations. Moreover, the benefit of laparoscopy is heightened in obese patients.^13^^,^^25^ Together these can offer significant technical advantages for the reversal of LIs in the increasingly obese surgical population.^13^ Open techniques for LI reversal require circumferential dissection from the level of the skin to the peritoneum prior to small bowel transection and anastomosis. Meticulous and time-consuming dissection through a thick anterior abdominal wall can be challenging as surgeons must pay close attention to avoid serosal injury or perforation of the underlying bowel.^26^^,^^27^ Whereas in laparoscopic LI reversal, small bowel transection and anastomosis has occurred prior to dissection of the two limbs of the loop ileostomy from the surrounding anterior abdominal wall, and thus damage to the afferent or efferent limbs is inconsequential to the anastomosis. Furthermore, intracorporeal anastomosis is technically feasible and safe, particularly in obese patients.^28^^,^^29^ One of the included studies limited their population to obese patients, however it was performed in the People's Republic of China where the definition of obese is a BMI of greater than 25.0 kg/m^2^.^13^ As such, data pertaining to the use of laparoscopic LI reversal in the North American obese population would add tremendous value to the current body of literature and perhaps accentuate the benefits observed in the present study.

Our review demonstrated a reduction in postoperative LOS after laparoscopic LI reversal. In North American, data on length of stay after loop ileostomy reversal has greatly varied over the last two decades.[30], [31], [32] A 2009 systematic review of 6107 patients published found a mean LOS of 5.1 days after ileostomy reversal.^33^ Concerns of prolonged ileus and high readmission rates may contribute to lengthier hospital stays, despite the lack of evidence supporting extended hospitalization for this relatively low-risk procedure.^34^^,^^35^ However, more recent reports support early discharge following ileostomy reversal, especially with the widespread implementation of enhanced recovery pathways (ERPs).^36^ Berger and colleagues reduced postoperative length of hospitalization to a median LOS of 2 days after implementation of an ERP, which was below the American College of Surgeons NSQIP median LOS of 4 days.^37^ Other studies have advocated for 23-h stay or day-case ileostomy closure, with no significant differences in risk of readmission or major postoperative morbidity (i.e., Clavien-Dindo (CD) Class 3–5), and have even demonstrated a potential reduction in minor postoperative morbidity (i.e., CD Class 1–2).^31^^,^[38], [39], [40] This being said, the included studies in the present review report LOS ranging from three to ten days, which may not reflect the recent trend towards shorter LOS after ileostomy reversal. Furthermore, though our meta-analysis found a statistically significant reduction in LOS after laparoscopic LI reversal compared to open reversal, a reduction by 0.39 days may not be of great clinical significance.

The strengths of the present systematic review and meta-analysis include the rigorous methodology, comprehensive risk of bias and GRADE assessments, and the quality of included data. The study limitations include the low certainty of evidence, small number of included studies, inclusion of only observational data increasing the risk of selection bias and residual confounding, and heterogeneity of the included studies. For example, in half of the patients in the laparoscopic group in the study by Sujatha-Bhaskar et al. anastomoses were performed extracorporeally.^14^ In the remainder of the studies laparoscopic LI reversal was performed via intracorporeal anastomosis.^12^^,^^13^^,^^15^ Additionally, variation in operative technique with regards to closure of the ileostomy site may have influenced observed rates of sSSIs. Randomized control trials have shown that purse-string closure of the stoma site leads to less sSSIs than conventional closure.[41], [42], [43], [44], [45] Next, the included studies do not report the time at which data from the different groups (laparoscopic versus open LI reversal) were collected. It is possible that the laparoscopic cases occurred later in the period and benefited from the addition of other interventions in contemporary ERPs, which might have contributed to the slight decrease in LOS in the laparoscopic group. Patient factors were fairly well matched between the laparoscopic and open LI groups and the relatively low overall risk of bias reduces the risk of selection bias. Nonetheless, patients who had undergone an open index operation were more likely to have an open LI reversal, which may have increased the technical difficulty of the LI reversal in the open group compared to the laparoscopic group due to adhesion formation.^46^ Ultimately, the major limitation of the present study is the limited number of included studies and patients. Further prospective study comparing laparoscopic and open LI could dramatically impact the results of this systematic review and meta-analysis as indicated by the GRADE assessment of meta-analyzed outcomes.

In summary, this study presents low quality evidence that laparoscopic LI reversal is a feasible approach that may reduce postoperative LOS and sSSI compared to open LI reversal without prolonging operative time. The reduction in LOS in the present study was relatively small. Future prospective comparative study is required to confirm the findings of the present review and clarify whether these represent meaningful clinical significance.

## Funding

This research did not receive any specific grant from funding agencies in the public, commercial, or not-for-profit sectors.

## Authors’ contributions

Conception and design of the study – All authors. Acquisition of data – McKechnie, Anpalagan. Analysis and interpretation of data – All authors. Drafting and revision of the manuscript – All authors. Approval of the final version of the manuscript – All authors.

**Supplementary Figure 1.** Risk of Bias in Non-randomized Studies of Interventions (ROBINS-I) assessment tool results per individual observational study.

**Supplementary Figure 2.** Risk of Bias in Non-randomized Studies of Interventions (ROBINS-I) assessment tool results grouped with all included studies.

**Supplementary Figure 3.** GRADE Certainty of Evidence Summary Table for meta-analyses.

## Declaration of Competing Interest

The authors declare that they have no known competing financial interests or personal relationships that could have appeared to influence the work reported in this paper.

## Appendix Supplementary materials

Image, application 1

Image, application 2

Image, application 3

Image, application 4
