# Supplementary material for: Laparoscopic versus open loop ileostomy reversal: A systematic review and meta-analysis
Source: Surg Pract Sci. 2023 Mar 23;13:100161. doi: 10.1016/j.sipas.2023.100161 (PMC11749981; doi:10.1016/j.sipas.2023.100161)
Supplement: Supplementary file 1 [file mmc1.pdf]

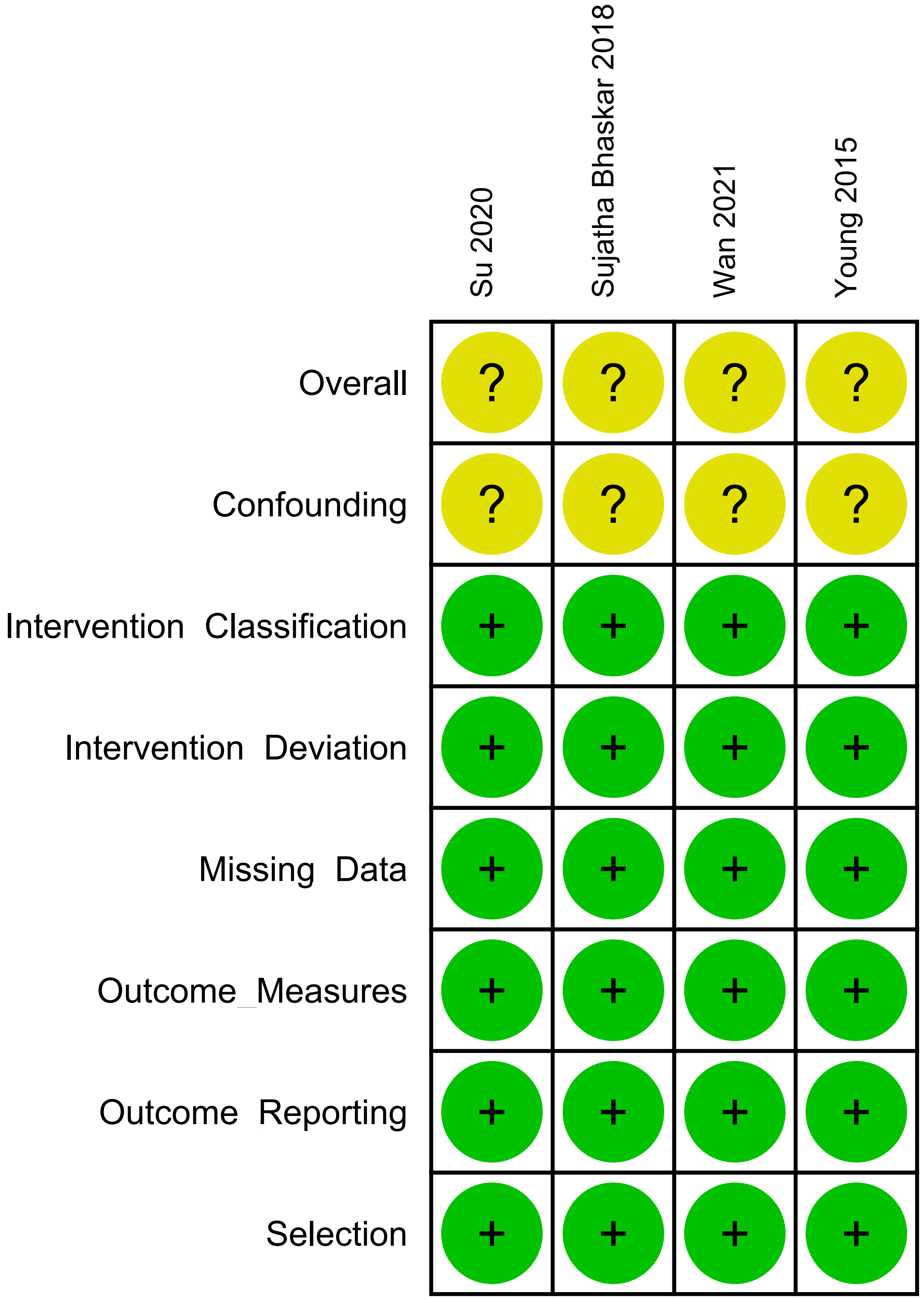

Su 2020
Sujatha Bhaskar 2018
Wan 2021
Young 2015
Overall
Confounding
Intervention Classification
Intervention Deviation
Missing Data
Outcome_Measures
Outcome Reporting
Selection
