# Supplementary figures and images for: Laparoscopic versus open loop ileostomy reversal: A systematic review and meta-analysis
Source: Surg Pract Sci. 2023 Mar 23;13:100161. doi: 10.1016/j.sipas.2023.100161 (PMC11749981; doi:10.1016/j.sipas.2023.100161)

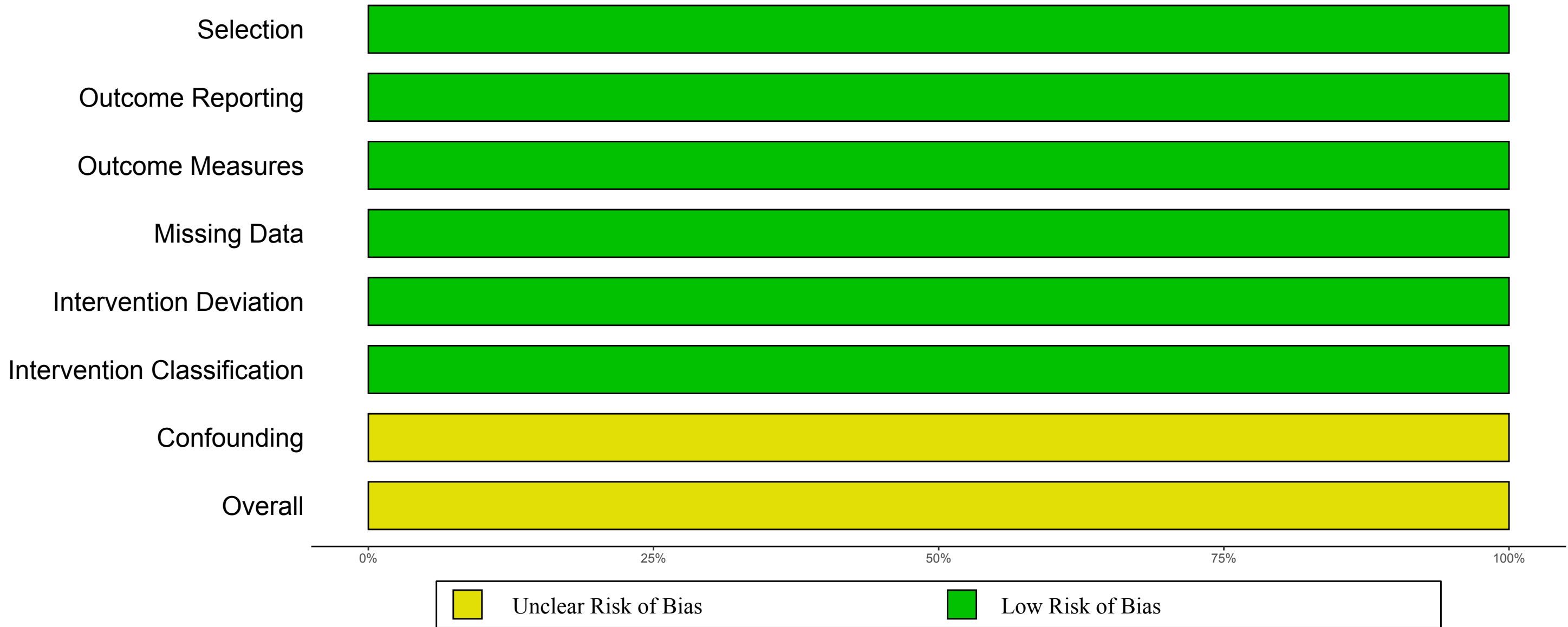

Supplement: Supplementary file 2 [file mmc2.pdf]
