# Supplementary material for: Laparoscopic versus open loop ileostomy reversal: A systematic review and meta-analysis
Source: Surg Pract Sci. 2023 Mar 23;13:100161. doi: 10.1016/j.sipas.2023.100161 (PMC11749981; doi:10.1016/j.sipas.2023.100161)
Supplement: Supplementary file 3 [file mmc3.pdf]

Certainty assessment							Summary of findings				
							Study event rates (%)			Anticipated absolute effects	
**Participants (studies) Follow-up**	**Risk of bias**	**Inconsistency**	**Indirectness**	**Imprecision**	**Publication bias**	**Overall certainty of evidence**	**With open loop ileostomy reversal**	**With laparoscopy loop ileostomy reversal**	**Relative effect (95% CI)**	**Risk with open loop ileostomy reversal**	**Risk difference with laparoscopy loop ileostomy reversal**
**Length of Stay**											
389 (4 observational studies)	not serious	not serious	serious[a]	serious[b]	none	⨁⨁◯◯ Low	176	213	-	The mean length of Stay was **5.6** days	MD **0.39 days fewer** (0.73 fewer to 0.04 fewer)
**Postoperative Morbidity (follow-up: 30 days)**											
389 (4 observational studies)	not serious	not serious	serious[c]	very serious[d]	none	⨁◯◯◯ Very low	36/176 (20.5%)	26/213 (12.2%)	**OR 0.62** (0.32 to 1.22)	205 per 1,000	**67 fewer per 1,000** (from 129 fewer to 34 more)
**Superficial Surgical Site Infection**											
389 (4 observational studies)	not serious	not serious	serious[c]	very serious[d]	strong association	⨁⨁◯◯ Low	13/176 (7.4%)	4/213 (1.9%)	**OR 0.22** (0.07 to 0.71)	74 per 1,000	**57 fewer per 1,000** (from 68 fewer to 20 fewer)
**Postoperative Ileus**											
256 (3 observational studies)	not serious	not serious	very serious[e]	very serious[d]	none	⨁◯◯◯ Very low	1/96 (1.0%)	5/160 (3.1%)	**OR 1.74** (0.28 to 10.91)	10 per 1,000	**8 more per 1,000** (from 7 fewer to 93 more)
**Operative Time**											
340 (4 observational studies)	not serious	serious[f]	serious[a]	very serious[d]	none	⨁◯◯◯ Very low	176	164	-	The mean operative Time was **106.9** minutes	MD **11.91 minutes more** (1.87 fewer to 25.7 more)
**Estimated Blood Loss**											
389 (4 observational studies)	not serious	serious[f]	serious[g]	serious[b]	none	⨁◯◯◯ Very low	176	213	-	The mean estimated Blood Loss was **69.1** mL	MD **2.09 mL more** (15.51 fewer to 19.7 more)

CI: confidence interval; **MD:** mean difference; **OR:** odds ratio

Explanations

a. Downgraded one point for significant differences in geographic location of study
b. Downgraded one point for small pooled sample sizes and large 95% confidence intervals
c. Downgraded one point for significant differences in indications for ileostomy formation and reversal
d. Downgraded two points for low event rates, small pooled sample sizes, and large 95% confidence intervals
e. Downgraded one point for differences in definitions of postoperative ileus across studies
f. Downgraded one point for I2 > 50%
g. Downgraded one point for imprecise and variable measures for recording EBL in the included studies
