# Supplementary material for: Laparoscopic versus open loop ileostomy reversal: A systematic review and meta-analysis
Source: Surg Pract Sci. 2023 Mar 23;13:100161. doi: 10.1016/j.sipas.2023.100161 (PMC11749981; doi:10.1016/j.sipas.2023.100161)
Supplement: Supplementary file 4 [file mmc4.docx]

**Supplemental Table 1.** Treatment characteristics of included studies (N, number of patients; SD, standard deviation; EBL, estimated blood loss; LOA, lysis of adhesions; LAR, low anterior resection; AR, anterior resection; STC, subtotal colectomy; IPAA, ileal pouch anal anastomosis; NR, not reported)

| Study | N total | Arm | N | Mean Duration of Ileostomy (days, SD) | Index Procedure Operative Approach | Type of Stoma Closure | Adjuvant Therapy | Mean Operative Time (mins, SD) | EBL (mL, SD) | LOA (%) |
| --- | --- | --- | --- | --- | --- | --- | --- | --- | --- | --- |
| Wan, 2021 | 60 | Laparoscopic | 48 | ﻿7.6 (6.8) | - | - | - | ﻿128.2 (41.7) | ﻿73.1 (91.3) | 42 (87.5) |
|  |  | Open | 12 | 13.0 (18.5) | - | - | - | ﻿142.5 (41.7) | ﻿94.2 (88.1) | 5 (41.7) |
| Su, 2020 | 64 | Laparoscopic | 30 | 7.8 (4.5-9.9)* | Laparoscopic- 30 (100) | - | Chemotherapy- 20 (66.7) | 88.0 (79.3-102.8)* | 20.0 (10.0-30.0)* | 21 (70.0) |
|  |  | Open | 34 | 7.5 (5.0-9.7)* | Laparoscopic- 34 (100) | - | Chemotherapy- 24 (70.6) | 77.5 (58.8-116.3)* | 20.0 (10.0-40.0)* | 18 (52.9) |
| Sujatha-Bhasker, 2018 | 132 | Laparoscopic | 82 | - | Open- 7 (8.5) Laparoscopic- 37 (45.0)  Robotic- 38 (46.3) | - | Chemotherapy- 27 (32.9) | 165.0 (41.1) | 24.95 (19.2) | 52 (63.4) |
|  |  | Open | 50 | - | Open- 19 (38.0) Laparoscopic- 14 (28.0)  Robotic- 17 (34.0) | - | Chemotherapy- 12 (24.0) | 140.7 (47.1) | 26.7 (32.4) | 28 (56.0) |
| Young, 2015 | 133 | Laparoscopic | 53 | 7.1 (15.0) | Open- 6 (11.3)  Laparoscopic- 17 (32.1)  Robotic- 30 (56.6) | Purse string- 47 (88.7)  Stapled with loose packing- 0 (0)  Left open- 6 (11.3) | Chemotherapy- 26 (49.1)  Radiation therapy- 14 (26.4) | 109.0 (45.0) | 31.0 (32.0) | 32 (60.4) |
|  |  | Open | 80 | 5.0 (5.9) | Open- 21 (26.3)  Laparoscopic- 45 (56.3)  Robotic- 14 (17.5) | Purse string- 62 (77.5)  Stapled with loose packing- 5(6.3)  Left open- 6 (7.5) | Chemotherapy- 23 (28.8)  Radiation therapy- 14 (17.5) | 93.0 (46.0) | 40.0 (81.0) | 21 (26.3) |

*=median (range)

**Supplemental Table 2.** Postoperative outcomes reported in included studies (N, number of patients; SD, standard deviation; LOS, length of stay; POI, postoperative ileus; AUR, acute urinary retention; UTI, urinary tract infection; sSSI, surgical site infection)

| Study | Arm | N | Mean LOS (days) | N Morbidity (%) | N POI (%) | N AUR (%) | N UTI (%) | N Anastomotic leak (%) | N sSSI (%) | N intraabdominal abscess (%) | N intestinal obstruction (%) | Median Cost (USD, range) |
| --- | --- | --- | --- | --- | --- | --- | --- | --- | --- | --- | --- | --- |
| Wan, 2021 | Laparoscopic | 48 | 10.3 (4.0) | 5 (10.4) | 1 (2.1) | - | - | 1 (2.1) | 2 (4.2) | - | - | 12224.6**^¶^ |
|  | Open | 12 | 10.8 (3.7) | 2 (4.2) | 0 | - | - | 0 | 2 (16.7) | - | - | 10156.8**^¶^ |
| Su, 2020 | Laparoscopic | 30 | 6.0 (6.0-7.0)* | - | 0 | 1 (3.3) | 0 | 0 | 2 (6.7) | 0 | - | - |
|  | Open | 34 | 7.0 (6.0-7.0)* | - | 0 | 0 | 0 | 1 (2.9) | 9 (26.5) | 0 | - | - |
| Sujatha-Bhasker, 2018 | Laparoscopic | 82 | 3.1 (2.4) | 9 (11.0) | 4 (4.9) | 4 (4.9) | 0 | 0 | 0 | - | - | 11017.5 (9820.3-13065.8) |
|  | Open | 50 | 3.4 (2.4) | 5 (10) | 1 (2.0) | 2 (4.0) | 0 | 0 | 0 | - | - | 10386.0 (9127.0-13855.0) |
| Young, 2015 | Laparoscopic | 53 | 5.3 (4.0) | 9 (16.9) | - | - | 3 (5.7) | 1 (1.9) | 0 | 2 (3.8) | 1 (1.9) | - |
|  | Open | 80 | 5.7 (4.6) | 17 (21.3) | - | - | 2 (2.5) | 4 (5.1) | 2 (2.5) | 5 (6.3) | 1 (1.3) | - |

*=median (range); **mean (no measure of central tendency); ¶converted from RMB to USD based on average 2021 exchange rate
